# Codevelopment and Deployment of a System for the Telemonitoring of Activities of Daily Living Among Older Adults Receiving Home Care Services: Protocol for an Action Design Research Study

**DOI:** 10.2196/52284

**Published:** 2024-02-29

**Authors:** Maxime Lussier, Mélanie Couture, Sylvain Giroux, Aline Aboujaoudé, Hubert Kenfack Ngankam, Hélène Pigot, Sébastien Gaboury, Kevin Bouchard, Carolina Bottari, Patricia Belchior, Guy Paré, Nathalie Bier

**Affiliations:** 1 Centre de recherche de l’Institut universitaire de gériatrie de Montréal Université de Montréal Montreal, QC Canada; 2 École de réadaptation, Faculté de médecine Université de Montréal Montréal, QC Canada; 3 Centre for Research and Expertise in Social Gerontology Integrated Health and Social Services University Network for West-Central Montreal Côte- Saint-Luc, QC Canada; 4 School of Social Work Université de Sherbrooke Sherbrooke, QC Canada; 5 Computer Science Department Faculty of Sciences Université de Sherbrooke Sherbrooke, QC Canada; 6 Department of Mathematics and Computer Science Université du Québec à Chicoutimi Chicoutimi, QC Canada; 7 School of Physical and Occupational Therapy Faculty of Medicine and Health Sciences McGill University Montreal, QC Canada; 8 Research Chair in Digital Health HEC Montréal Montréal, QC Canada

**Keywords:** action design research, protocol, activities of daily living, older adults, cognitive deficits, telemonitoring, public health care system, home care services

## Abstract

**Background:**

Telemonitoring of activities of daily living (ADLs) offers significant potential for gaining a deeper insight into the home care needs of older adults experiencing cognitive decline, particularly those living alone. In 2016, our team and a health care institution in Montreal, Quebec, Canada, sought to test this technology to enhance the support provided by home care clinical teams for older adults residing alone and facing cognitive deficits. The Support for Seniors’ Autonomy program (SAPA [Soutien à l’autonomie des personnes âgées]) project was initiated within this context, embracing an innovative research approach that combines action research and design science.

**Objective:**

This paper presents the research protocol for the SAPA project, with the aim of facilitating the replication of similar initiatives in the future. The primary objectives of the SAPA project were to (1) codevelop an ADL telemonitoring system aligned with the requirements of key stakeholders, (2) deploy the system in a real clinical environment to identify specific use cases, and (3) identify factors conducive to its sustained use in a real-world setting. Given the context of the SAPA project, the adoption of an action design research (ADR) approach was deemed crucial. ADR is a framework for crafting practical solutions to intricate problems encountered in a specific organizational context.

**Methods:**

This project consisted of 2 cycles of development (alpha and beta) that involved cyclical repetitions of stages 2 and 3 to develop a telemonitoring system for ADLs. Stakeholders, such as health care managers, clinicians, older adults, and their families, were included in each codevelopment cycle. Qualitative and quantitative data were collected throughout this project.

**Results:**

The first iterative cycle, the alpha cycle, took place from early 2016 to mid 2018. The first prototype of an ADL telemonitoring system was deployed in the homes of 4 individuals receiving home care services through a public health institution. The prototype was used to collect data about care recipients’ ADL routines. Clinicians used the data to support their home care intervention plan, and the results are presented here. The prototype was successfully deployed and perceived as useful, although obstacles were encountered. Similarly, a second codevelopment cycle (beta cycle) took place in 3 public health institutions from late 2018 to late 2022. The telemonitoring system was installed in 31 care recipients’ homes, and detailed results will be presented in future papers.

**Conclusions:**

To our knowledge, this is the first reported ADR project in ADL telemonitoring research that includes 2 iterative cycles of codevelopment and deployment embedded in the real-world clinical settings of a public health system. We discuss the artifacts, generalization of learning, and dissemination generated by this protocol in the hope of providing a concrete and replicable example of research partnerships in the field of digital health in cognitive aging.

**International Registered Report Identifier (IRRID):**

RR1-10.2196/52284

## Introduction

### Background

In Canada, it is estimated that 2 million people, or 5.4% of the total population, are active users of publicly funded home care services, with more than half of them being aged ≥65 years [1,2]. Home care is considered a priority by the Canadian government and its provincial counterparts [3]. In the province of Quebec, a predominantly French-speaking province in Eastern Canada, the Ministry of Health and Social Services has promoted aging in place since 2003 through a policy known as “Home Support: Always the Option of Choice” (“Chez soi: Le premier choix”) [4]. Despite this policy, 40% of older adults in Quebec living with disabilities report unmet home care needs [2], which has been attributed to factors such as the lack of funding, difficulties in recruiting and retaining home care staff, and the increase in care needs in an aging population [5,6]. These challenges, exacerbated by the COVID-19 pandemic, have led to urgent calls in Canada as well as in other countries to better support home care [6,7].

The presence of cognitive deficits is an important risk factor for the loss of functional autonomy [8,9] and is associated with important needs for home care support services [6]. Older adults with cognitive decline, such as those diagnosed with Alzheimer disease, are progressively dependent on others for both instrumental and basic activities of daily living (ADLs), which increases the stress and burden on caregivers (families and relatives) and social and health care services [8,9]. Everyday memory losses and difficulties identifying and recognizing their own limitations affect the ability to recall and report on events, including how they manage their ADLs [10,11]. These individuals may also put themselves at risk by engaging in behaviors that are dangerous to their health and safety, in particular when they live alone (eg, falls, kitchen fires, and medication noncompliance) [12].

Therefore, progressive cognitive decline and its impact on ADLs limit the ability of health and social services to rely on these patients to better understand their home care service needs. This uncertainty can significantly affect intervention plans and resource management [13,14]. On the one hand, this may lead to the costs of some care recipients’ needs not being covered by public care services because of a lack of supporting evidence; on the other hand, some services may be offered although they are unnecessary because of clinicians’ need to create contingencies to address potential risks [15,16].

In this context, we propose that a key strategy to better understand the needs of older adults with cognitive decline and optimize available human and financial resources lies in the use of digital health technologies, in particular telemonitoring of instrumental and basic ADLs—hereafter referred to as *ADL telemonitoring*.

### Telemonitoring of ADLs

ADL telemonitoring is a form of remote monitoring and, as such, a component of telehealth. It is generally used to acquire proxy information about several factors or outcomes relevant to the home care support of older adults, such as functional independence, cognitive state, mobility, risk of falls, and urinary tract infections [13,14,17,18]. The information is based on data collected via several sensors, such as wearables, ambient sensors (eg, motion detectors and contact sensors), radiofrequency identification, and GPS [19]. The resulting profile of the person is based on information collected regarding their behavior in the home over the course of one or several days, such as entering and leaving a room, opening and closing kitchen cupboards, home entrances and exits, and the use of small electrical appliances. Various algorithms can be used to recognize and classify ADLs, including hidden Markov models, linear discriminant analysis, support vector machines, artificial neural networks, and adaptive network fuzzy inference system [20].

ADL telemonitoring can be used in the context of naturalistic scenario-based assessments in which participants are invited to a research laboratory apartment designed to simulate real-life situations. There, participants are usually asked to perform several scripted ADLs, such as cooking a meal, cleaning the apartment, or booking a reservation [21-24].

ADL telemonitoring can also be used in the context of real-life assessments in which participants are asked to continue their daily routine while sensors are installed directly in their homes to collect data on sleeping habits, meal preparation, hygiene, mobility in the home, periods outside the home, and so on [25,26]. Over time, deviations from previous patterns can be detected and used to assess the ability of an older adult to live independently in the community and detect potential future critical situations [27-30].

Thus, ADL telemonitoring holds great promise to facilitate better understanding of the needs of older adults with cognitive decline, in particular those living alone, in terms of ADL functioning and home care service requirements. Indeed, research in the area of ADL telemonitoring has been expanding over the last 20 years. In a recent umbrella review, Tannou et al [31] identified over 191 experiments in the field and 17 reviews published to date. Despite this dynamism, there is still little available evidence on the effectiveness of ADL telemonitoring and strategies for integrating it successfully into health care systems [31]. Most studies have reported data on prototypes, but very few large-scale deployment studies have been conducted. Therefore, there is a pressing need for more in-depth studies on this subject.

### Context and Aim

In 2016, the authors were approached by a health institution in the city of Montreal, Quebec, Canada, more specifically by the director of the *Support for Seniors’ Autonomy* program (*Soutien à l’Autonomie des Personnes Âgée*s in French; SAPA). SAPA programs aim to support older adults and meet their physical, psychological, and gerontopsychiatric needs both in the community and during hospital stays. The director was searching for innovative ways to support home care clinical teams to better address the needs of older adults with cognitive deficits who live alone. The directorate was also looking for solutions to optimize the use of limited human and financial resources to offer the right service at the right moment. Our team and the top management team decided to engage in a joint research project on ADL telemonitoring, which became the “SAPA Project.” The global objectives of the SAPA project were to (1) codevelop an ADL telemonitoring system that addresses the needs of all key stakeholders, (2) deploy the system in a real-world clinical setting and identify the specific use cases, and (3) identify factors that could contribute to ensuring its long-term use in a real-world setting. To foster the project’s success, we engaged with all key stakeholders involved in public home care services for older adults with cognitive deficits. We considered that, to ensure the optimal use and adoption of technologies by the health care community, it would be crucial to identify existing solutions or create novel solutions that address the specific needs of all key stakeholders, including the care recipients themselves [32]. To do so, stakeholders had to be involved in every step of the project to align their needs with the technology [33].

Considering the context of the SAPA project, the use of an action design research (ADR) approach was identified as crucial. ADR provides a framework for developing practical solutions to complex problems encountered in a specific organizational setting. It is an iterative process that involves collaborations among researchers, practitioners, and stakeholders in designing [34], deploying, and evaluating interventions that improve the outcomes of a particular problem or issue [35]. This four-stage research process includes (1) problem formulation; (2) building, intervention, and evaluation; (3) reflection and learning; and (4) formalization of learning. ADR proposes the use of multiple cycles of development leading to cyclical repetitions of stages 2 and 3. Carried out in collaboration with the stakeholders (codevelopment process), these cycles allow for the refinement of the technology and related interventions to be implemented subsequently in a real-world setting. The SAPA project underwent 2 iterative technological design cycles entitled “alpha” and “beta.” The alpha cycle took place from early 2016 to mid 2018, and the beta cycle took place from late 2018 to late 2022.

A key feature of the SAPA project was its novel methodological approach. In this paper, we present the SAPA research protocol and its various codevelopment and deployment phases for the 2 cycles of ADR. To our knowledge, this represents the first detailed publication of a methodological framework in this field of research. Taking the initiative to share a research design makes it possible to be more transparent about the methodologies used as well as provide an in-depth explanation of the process so that it can be reproduced in the future. It also provides a description of a concrete example of research partnerships in the field of digital health in cognitive aging.

**Figure 1:**
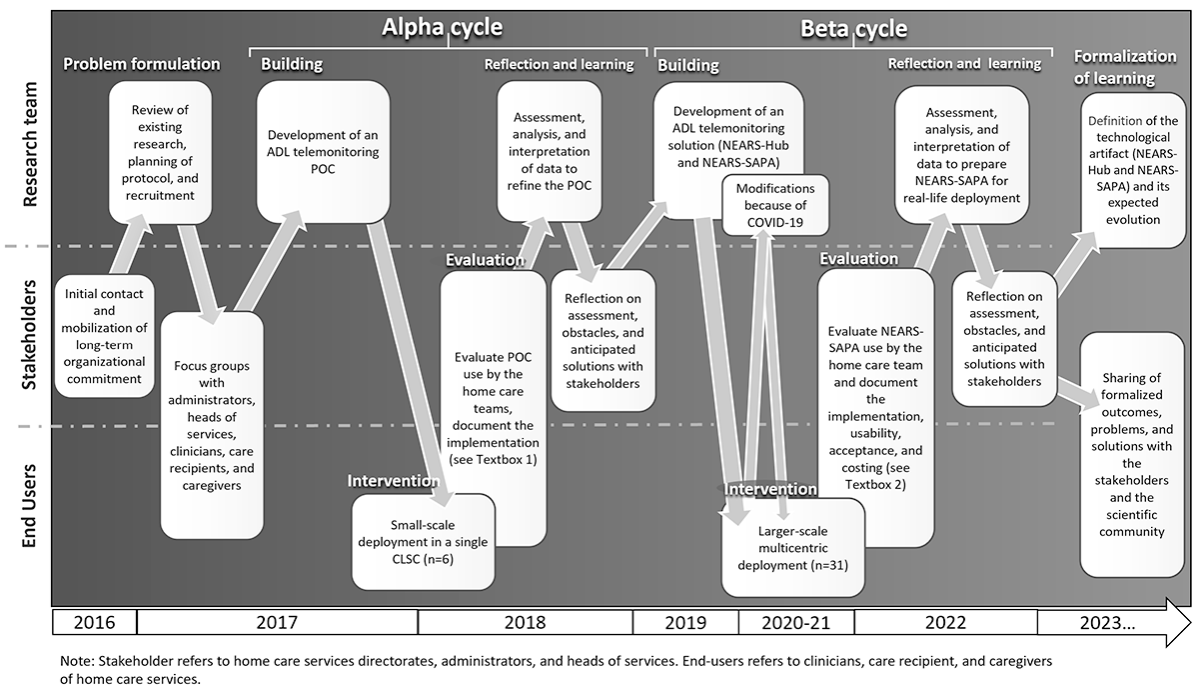
Summary of action design research protocol (based on the studies by Sein et al and Schacht et al). ADL: activity of daily living; CLSC: local community services center; NEARS-Hub: Innovative Easy Assistance System–Hub; NEARS-SAPA: Innovative Easy Assistance System–Support for Seniors’ Autonomy program; POC: proof of concept.

In the following sections, we will present each cycle and stage of the project, which involve multiple research methods as recommended by action research methodologists [36]. Specific methodological details are presented in Multimedia Appendix 1. Figure 1 [35,37] presents an overview of the project’s timeline and key elements of each cycle and stage.

## Alpha Cycle

### Methods

#### Background

The SAPA project aimed to provide an in-depth understanding of the potential of ADL telemonitoring in the specific context of the Canadian public health care system, particularly in the province of Quebec. This province has an estimated population of 8.3 million residing in 18 administrative areas referred to as health regions. For a given health region, all public health and social services institutions are networked into integrated health and social services centers (CISSSs [*Centre intégré de santé et de services sociaux*]) or integrated university health and social services centers (CIUSSSs [*Centre intégré universitaire de santé et de services sociaux*]) when located in an area where a university offers an undergraduate medical training program or has an institute in the social services field. These administrative entities are responsible for delivering care and services to the population of an assigned territory via hospitals, residential and long-term care establishments, rehabilitation centers, child and youth protection centers, and local community service centers (CLSC [*Centre local de services communautaires*]). CLSCs offer frontline health and social services, including home care, through dedicated programs.

The alpha cycle of the SAPA project was conducted within the clinical services of the CLSC of one CIUSSS in the city of Montreal referred here as CIUSSS 1.

#### Problem Formulation

The alpha cycle started with a problem formulation stage aimed at conceptualizing the research problem, securing long-term organizational commitment, and clarifying roles and responsibilities with stakeholders. This stage combined theoretical and experiential knowledge informed by real-life practices [35]. As previously mentioned, the initial problem (ie, the need to support home care clinical teams in meeting the needs of older adults with cognitive difficulties living alone in the community) was brought to the research team by the health institution.

To arrive at a mutual understanding of the problem and prioritize potential technological solutions at the start of the project in late 2016, a descriptive qualitative method was followed [38]. More specifically, individual interviews and focus groups were conducted with key stakeholders (administrators, heads of services, clinicians, older adults, and caregivers; n=23 in total) of 1 specific sector covered by a CLSC of CIUSSS 1 (for more details, see Multimedia Appendix 1, section 1, as well as the study by Couture et al [34]). This sector was chosen by the head administrator as the most appropriate site for project deployment. Participants were questioned about the leverages of and obstacles to the home care of older adults and technology use within this context. At that time, neither the researchers nor the clinical team had a specific technology in mind to address the problem. However, to support the reflection process, several technological solutions were presented to the clinical teams. These included assistive devices and monitoring technologies such as smart home sensors, smart calendars, pill distributors, serious games, and stove safety devices.

One of the main findings was that administrators and clinicians thought that monitoring technologies could help them obtain more objective and reliable information to support their decision-making process to develop an adapted intervention plan with the care recipients [34,39]. More precisely, they wanted a technology to support their assessment and management of the risks involved in maintaining care recipients at home as long as possible. A second finding was that stakeholders insisted on avoiding the use of cameras or microphones to preserve intimacy and confidentiality [34,39]. They also wanted to avoid wearable technologies as older adults with cognitive deficits would likely forget to wear them. Finally, it was mentioned that an unobtrusive technology requiring little involvement on the part of the older adults would most likely be successfully implemented.

Considering these expectations, ADL telemonitoring seemed to be the most promising technology to fully address the needs expressed by all. At the time of the study, there was no such system available on the market and ready to be deployed in a home care setting. Therefore, we resolved to design a system specifically for this research project based on our previous work [13,24,40-43]. With the stakeholders’ approval of the project scope and vision, we secured a long-term organizational commitment as well as funding for the subsequent phases.

#### Building, Intervention, and Evaluation

##### Overview

In the second stage of ADR, the initial design of the technological artifact is generated using the problem framing adopted in the first stage [35]. The building, intervention, and evaluation phase, which is an iterative process conducted in the target environment, involves building the technological artifact, intervening in the organization, and evaluating the outcomes. The product of this stage is the design of the artifact.

At this point in the alpha cycle, the overall target and goal of knowledge generation was to design and create an ADL telemonitoring system to support clinicians’ decision-making process related to risk management. As is often the case in IT-dominant artifact creation, we opted to develop a proof of concept (POC) that could be quickly developed and tested in the homes of a small sample of older adults receiving home care services (ie, alpha cycle). POC versions are formative and can help validate several anticipated and unanticipated outcomes of technology use and deployment [35]. Early designs serve as lightweight interventions in a limited organizational context.

##### Building

**Figure 2:**
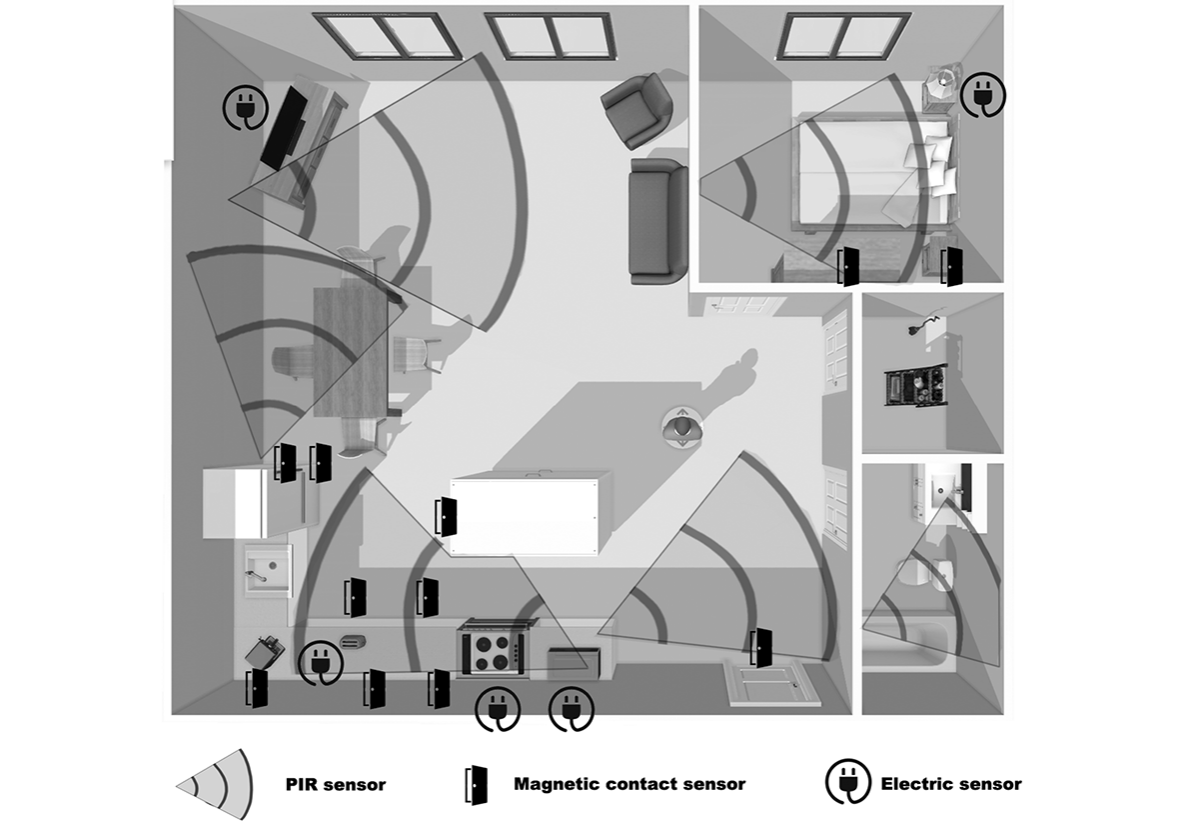
Example of sensor placement in the home. PIR: passive infrared.

For the POC, we used a lightweight sensor infrastructure comprising 4 nonintrusive, low-cost, wireless devices similar to the one deployed in the study by Caroux et al [44]. With their consent, sensors were deployed in each participating patient’s home: passive infrared sensors, magnetic contact sensors, smart electric switches, and water sensors. Sensors were paired with an Internet of Things device, the VeraPlus Z-Wave Home Controller, which served as the data collection point (ie, a controller). Sensors were positioned in each home so as to capture data on ADLs, including sleeping, cooking, showering, and leaving the home (Figure 2). Multimedia Appendix 1, section 2, provides more details on each sensor’s role.

Algorithms using an inference system and finite state machine computation models were developed to monitor ADLs such as sleep, periods outside the home, cooking, hygiene, and general activity levels in the home (for more details, see the study by Lussier et al [45]). From these algorithms, it was possible to relay graphical information about the daily habits of the individual to clinicians to support their decision-making process. The information was summarized on a 3-page PDF report sent directly to the clinicians.

##### Intervention

In the intervention phase, the POC needs to be used firsthand by practitioners from the real-life setting for which it is designed. This participatory process provides opportunities for end users to influence and guide the design.

In early 2017, the POC was presented to clinicians and managers in team meetings in the identified testing sector. Clinicians were invited to identify older home care recipients for whom they thought ADL telemonitoring would be useful. Clinicians who wished to participate filled out a request form about their needs and the care recipient they identified. Complex cases were discussed with the research team to ensure that the needs of the clinician were within the scope of ADL telemonitoring. Recruitment procedures are detailed in Multimedia Appendix 1, section 3.

##### Evaluation

###### Overview

The general objective of the alpha cycle was to document the feasibility of using ADL telemonitoring in real-life settings and quickly iterate the POC. More specifically, the objectives pursued were to (1) describe the characteristics of the participants and their demands for telemonitoring, (2) describe the use and impact of the POC by the home care teams, and (3) document the facilitators of and barriers to POC use.

**Figure 3:**
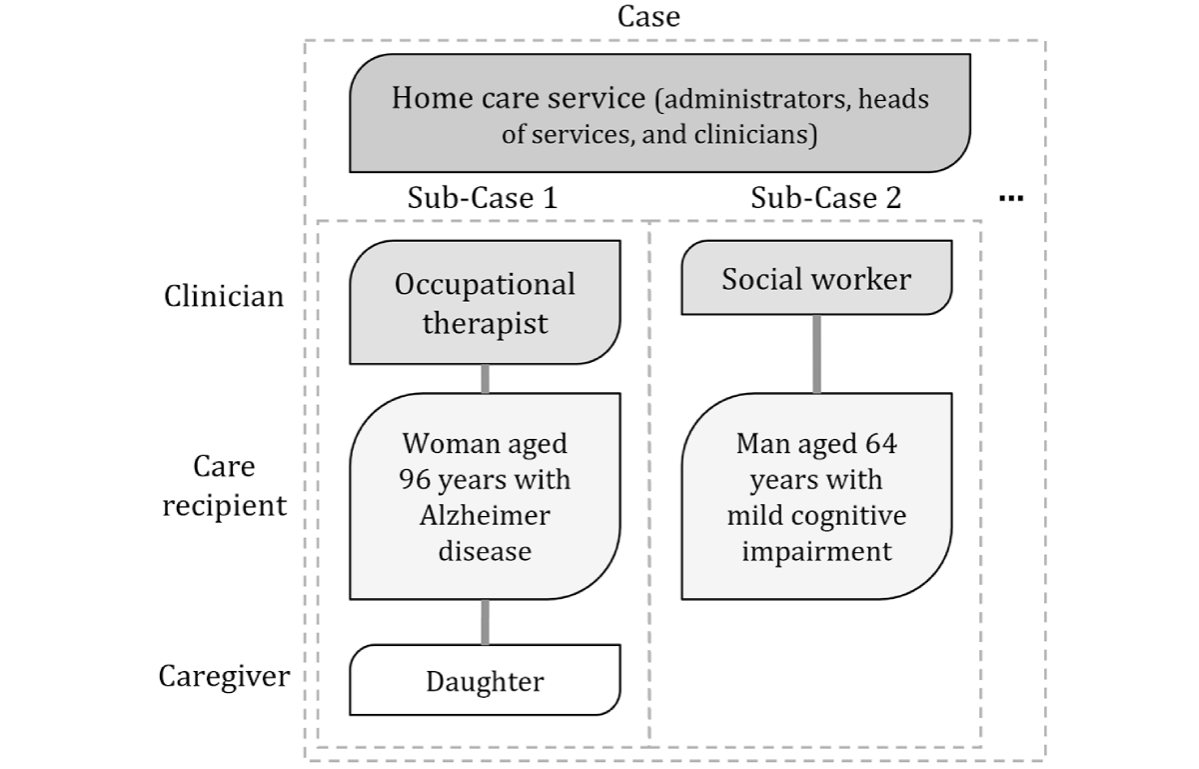
Diagram of embedded case study.

To achieve these objectives, a mixed embedded single-case study design was used [46]. Case studies are relevant when the research question requires a comprehensive description of a social phenomenon [46], as is the case here in studying the perspectives of different stakeholders on the feasibility of using technology. The single case was the home care division of CIUSSS 1. The 6 subunits consisted of care recipients (n=6); their clinicians (n=8; 2 were replaced during the project) from home care services; and, when possible, a family caregiver (n=2; Figure 3). These subunits made it possible to shed light on the personalized trajectories of each care recipient as well as the intended and actual use of the system in relation to these trajectories. Multimedia Appendix 1, section 6, provides more details on the subunits and data analysis methods. A mix of quantitative and qualitative data was collected. Textbox 1 provides an overview of the data collection process in the alpha cycle.

**Overview of the alpha cycle data collection, sample, measurements, and variables in the evaluation stage.**

**Telemonitoring request form filled out by clinicians and with follow-up questions via phone interviews**

- Facilitators of and barriers to home care of care recipients
- Demands for telehealth technology

**Characteristics of clinicians (n=8) and care recipients (n=6)**

- For clinicians: profession
- For care recipients only: age, sex, years of schooling, Mini-Mental State Examination, Montreal Cognitive Assessment, Multiclientele Assessment Tool, Functional Autonomy Measurement System profile, metadata on the home care service received, cognitive evaluation (Rey Auditory Verbal Learning Test; Mini-Geriatric Depression Scale; and Delis-Kaplan Executive Function System Trail Making test, Stroop test, and Tower Test), and Instrumental Activity Profile

**Initial individual interviews with care recipients (n=6) and caregivers (n=2)**

- Facilitators of and barriers to home care of care recipients
- Anticipated leverages of and obstacles regarding telemonitoring deployment in home care

**Total of 3 follow-up focus groups with clinicians after proof of concept installations (n=8)**

- Observed facilitators of and barriers to telemonitoring deployment
- Use case, impacts, and general satisfaction regarding deployment

**Postdeployment focus groups with administrators (n=2), heads of services (n=5), and clinicians (n=8)**

- General facilitators of and barriers to telemonitoring deployment and sustainability
- Impacts of and general satisfaction with telemonitoring

###### Characteristics of Participants and Demands for ADL Telemonitoring

With the care recipients’ consent, their medical history was extracted, including cognitive screening scores as well as needs for home care support, sociodemographic information, and the quantity of home care services required. Metadata were also extracted from the medical records about each service provided by the health network for each care recipient (ie, type of act and its time and duration). Finally, to obtain a more in-depth cognitive profile of each care recipient, a cognitive evaluation, as well as an evaluation of ADL performance, was completed by a research assistant. Multimedia Appendix 1, section 6, provides more details.

Clinicians who identified a care recipient and wished to participate filled out a request form detailing their demands for telemonitoring, the reasons that led them to request remote monitoring for this care recipient, and which ADL they would like to obtain more information about via telemonitoring. Through interviews, they were also asked about facilitators of and barriers to home care for this specific care recipient. After receiving the form, a research assistant called the clinician to provide any further details or clarifications as needed.

###### Use and Benefits of ADL Telemonitoring

During the follow-up focus groups, clinicians were asked how telemonitoring was used, for which ADL, and how it influenced their decision-making process regarding the recipients’ care plan. They were also asked about their satisfaction, whether it was relevant to continue telemonitoring for each care recipient, and why.

Finally, during postdeployment focus groups, administrators, heads of services, and clinicians were asked about their general satisfaction with telemonitoring as well as the benefits it had for clinical practice.

###### Facilitators of and Barriers to ADL Telemonitoring Deployment

During the initial interviews, care recipients and caregivers were asked whether they foresaw any barriers to and facilitators of the deployment of the ADL telemonitoring system. In addition, to document their perceived needs, they were asked to describe ADLs that were difficult for them to perform, the current help they received, and their unmet care support needs.

In follow-up focus groups, clinicians were asked about the facilitators and obstacles they encountered during deployment.

During postdeployment focus groups, participating clinicians and managers were asked about elements that could promote or hinder deployment of the ADL telemonitoring system in their organization as well as their thoughts regarding the sustainability of this type of system.

Finally, throughout the study, data on several additional factors to be considered were collected: occurrence of adverse events; clinician turnover; reason for care recipient’s withdrawal, relocation, or hospitalization; or cause of death.

#### Ethical Considerations

The alpha cycle was approved by the Centre de recherche de l’Institut universitaire de gériatrie de Montréal research ethics board (CER VN 16-17-22). All participants (ie, care recipients, clinicians, caregivers, and managers) provided informed and written consent before taking part in the data collection process. Information that could potentially identify participants was securely stored in a locked storage unit or protected by passwords. Whenever possible, data were deidentified through codification. Participants did not receive any monetary compensation.

### Results

#### Overview

The *Reflection and learning* stage of ADR mirror a conventional *Results* section, entailing the analyses and interpretation of the data collected during the evaluation phase. The alpha cycle aimed to develop and deploy the POC in a real-life context as well as quickly reveal anticipated and unanticipated outcomes regarding technology use and deployment. In the following sections, we synthesize the outcomes for each of the 3 objectives. Multimedia Appendix 1, section 5, provides more details on the outcomes.

#### Characteristics of Participants and Demands for ADL Telemonitoring

The system was deployed in the homes of 4 care recipients with severe cognitive deficits. They all lived alone, and half (2/4, 50%) had family caregivers. Participating clinicians included 3 occupational therapists, 3 nurses, and 2 social workers.

During these interviews, social and health care professionals expressed the need for monitoring data to support their clinical decision-making process. More precisely, they wanted access to technology that could help assess and manage the risks involved in maintaining care recipients at home. In other words, their aim was to gain a better understanding of how care recipients were functioning in their homes. This information would support their intervention plan and help determine which services should be put in place.

#### Use and Benefits of ADL Telemonitoring

The evaluation stage revealed that the ADL telemonitoring system was used by clinicians to collect additional and reliable data about the home care recipient’s ADL routine (for more details, see the study by Lussier et al [15]). In accordance with the demands for ADL telemonitoring, this technology made it possible to gain a better understanding of how care recipients were functioning in their homes, providing an objective measure that complemented other subjective clinical evaluations. More precisely, it was used to confirm or refute hypotheses before developing a comprehensive intervention plan. It was also used to facilitate discussions with the older adults and their families based on consensual information

Overall, clinicians described the monitoring technology as a useful tool for allocating home care services that corresponded to the needs of care recipients and optimizing their autonomy and safety [15].

#### Facilitators of and Barriers to ADL Telemonitoring Deployment

Overall, the alpha cycle presented several encouraging results regarding the POC’s perceived usefulness and reported satisfaction, as well as the feasibility of its deployment in SAPA teams operating in the field [39]. Although the prototype was successfully deployed in 4 care recipients’ homes, several obstacles were encountered. In total, 3 main obstacles were identified: the characteristics of some participants, burdensome evaluation protocol, and maturity level of the POC.

First, the technology used for the prototype was suboptimal for some care recipients’ profiles. More specifically, a concern was expressed that sensors would exacerbate symptoms of a paranoid personality disorder with invasive thoughts, and consequently, this care recipient was not included in further testing. In addition, the usefulness of the sensors for care recipients presenting important mobility challenges was questioned. For instance, these care recipients performed very few ADLs by themselves and moved around their units in a wheelchair—which decreased monitoring efficiency and accuracy.

Second, the alpha cycle protocol allowed us to better understand the population followed by the CLSC and refine the protocol for evaluating care recipients’ characteristics. In fact, our original protocol was too long, formal, and confronting for many care recipients, increasing the risk of a refusal to participate in the study or withdrawal. Thus, it was decided that formal evaluations with care recipients would be reduced as much as possible in subsequent cycles.

Overall, the feedback showed that the prototype was not ready for wider deployment—the installation took too long, data processing was too slow, output analysis was complex and time-consuming, and it was mostly done by hand, among other issues. Finally, despite the lack of real danger, it was decided that water sensors were to be avoided in the future as they were the only type of sensor that generated anxiety in almost all care recipients because of their concerns regarding having wires near water.

## Beta Cycle

### Methods

#### Building, Intervention, and Evaluation

##### Overview

Following the alpha cycle stage and deployment of the POC, CIUSSS 1 was interested in participating in a second cycle of development with a wider deployment. In addition, during the alpha cycle, 2 other institutions from the Greater Montreal area (CIUSSS 2 and CISSS 3) manifested their interest in using the ADL telemonitoring system, and researchers from our team met with managers and clinicians from these institutions. They identified the needs and challenges of home care for older adults with cognitive deficits that were very similar to those encountered by CIUSSS 1. As such, the beta cycle was conducted with CIUSSS 1, CIUSSS 2, and CISSS 3 without repeating the problem formulation stage. Furthermore, a second problem formulation stage was deemed nonrelevant as the alpha cycle results did not indicate a need to refine our understanding of the context.

The general goals of the beta cycle were to further improve the ADL telemonitoring system based on experience gathered during the POC and deploy this newer version on a wider scale to further document specific use cases and factors that could contribute to ensuring its long-term use in a real-world setting. As the COVID-19 pandemic occurred as deployment for the beta cycle was just starting, many adjustments to the research protocol and monitoring system had to be made. These are summarized in the following sections and detailed in Multimedia Appendix 1, section 6.

##### Building

Between 2018 and 2019, a blitz of technological developments was undertaken to develop an edge computing based on a distributed architecture that offers more flexibility for integrating new technologies with high performance in data ingestion than the POC developed in the alpha cycle. Details of the system optimization can be found in Multimedia Appendix 1, section 7.

Organized into software modules, the system centralizes data encryption, storage, computation, communication, and system robustness on a single low-power, low-cost computing device. This framework, called the Innovative Easy Assistance System (NEARS)–Hub, integrates nonintrusive sensors in the smart home [47].

Several features were added to build on the alpha cycle learning and address limitations. First, the deployment of sensors was optimized, which not only simplified the installation but also minimized the technicians’ presence in the patients’ homes. The latter was a particular source of concern for both clinicians and care recipients.

Second, to facilitate rapid data collection, the VeraPlus Z-Wave Home Controller was replaced with a Raspberry Pi 3 linked with a USB Z-Wave controller, offering several options for local data preprocessing and a number of other improvements, such as the automation of collection, maintenance, and data processing; local data storage; and automation of local backups to compensate for service interruptions.

Third, 2 additional types of sensors were integrated into the ecosystem: a heavy-appliance smart electric switch to monitor oven use and pressure mattress sensors that could be added to the bed or couch.

Fourth, a major achievement was compliance with the safety and confidentiality standards required by the Quebec Ministry of Health and Social Services.

Fifth, a web-based user interface supporting advanced data visualization and comparison tools, called NEARS-SAPA, was designed for clinicians, enabling them to monitor (1) 5 significant ADLs (ie, sleep, eating, mobility, activity, and hygiene), (2) relevant object use (eg, fridge, television, and coffee maker), and (3) room occupancy. As a result, the platform provided a broad overview of the ADLs performed by each care recipient as well as some specific information regarding each ADL (eg, the care recipient’s sleeping schedule). To compensate for both the inability of research staff to train clinicians because of the pandemic and clinicians’ increased workload, the research team simplified reporting, sending a data summary electronically in PDF highlighting the trends and changes in ADLs for each care recipient every 2 months.

Finally, to reduce in-person home visits related to fixing technical problems, a modular and component-based IT infrastructure was built into the NEARS-Hub to solve as many maintenance issues as possible remotely.

##### Intervention

Strategies based on the alpha cycle were put in place to improve deployment (see Multimedia Appendix 1, section 8, for details). With the involvement of managers from each health institution, it was decided that the project and its opportunities would be presented during regularly scheduled meetings of each targeted profession. Coordinators from different professional teams were asked to promote the project to their colleagues. Although it was not considered an exclusion criterion, clinicians were alerted to poor acceptance and possible adverse outcomes of ADL telemonitoring in individuals with paranoid disorders and extensive mobility challenges.

##### Evaluation

###### Overview

For the beta cycle, the objectives were as follows: (1) to describe the characteristics of the participants and their demands for telemonitoring, (2) to describe the use and benefits of the ADL telemonitoring system on a larger scale, (3) to document facilitators of and barriers to its deployment, (4) to evaluate the usability and acceptance of the system, and (5) to estimate the costs related to system deployment and use. To respect COVID-19 sanitary guidelines, interviews and evaluations were conducted through telephone.

Textbox 2 provides an overview of the data collection process used during this cycle. Details can also be found in Multimedia Appendix 1, section 9.

**Overview of the beta cycle data collection, sample, measurements, and variables in the evaluation stage.**

**Telemonitoring request form filled out by clinicians, with follow-up questions via phone interview**

- Facilitators of and barriers to home care of care recipients
- Demands for telehealth technology

**Characteristics of clinicians (n=31), care recipients (n=31), and caregivers (n=9)**

- For all: demographics
- For care recipients only: Mini-Mental State Examination, Montreal Cognitive Assessment, Multiclientele Assessment Tool, and Functional Autonomy Measurement System profile

**Before deployment—individual interviews with clinicians (n=31), care recipients (n=8), and caregivers (n=9)**

- Facilitators of and barriers to home care of care recipient
- Anticipated leverages of and obstacles to telemonitoring deployment in home care
- Demands for telehealth technology

**During deployment—follow-up individual interviews with clinicians (n=31) every 4 months**

- Observed facilitators of and barriers to telemonitoring deployment
- Use case, impacts, and general satisfaction regarding telemonitoring

**Questionnaires for clinicians (n=23)**

- 10 in-house questions on use
- 7 questions from the System Usability Scale on usability
- 11 questions from the technology acceptance model–2 on acceptance

**Questionnaires for care recipients (n=23)**

- 3 in-house questions on telemonitoring perceived usefulness
- 8 in-house questions on telemonitoring acceptance

**After deployment—focus groups with clinicians (n=8)**

- General facilitators of and barriers to telemonitoring deployment and sustainability
- Impacts of and general satisfaction with telemonitoring

**After deployment—individual interviews with administrators (n=2)**

- General facilitators of and barriers to telemonitoring deployment and sustainability
- Impacts of and general satisfaction with telemonitoring

**Estimate of direct, indirect, and intangible costs related to telemonitoring**

- Material costs
- Time invested
- Adverse outcomes reported

###### Characteristics of Participants and Demands for ADL Telemonitoring

For subunit characteristics, we used the same measurements as for the alpha cycle but dropped the cognitive and functional evaluations because of sanitary constraints and adverse reactions reported by clinicians and caregivers from the alpha cycle. For clinicians, age, sex, profession, affiliation, and years of experience in home care services were collected (self-reported). As in the alpha cycle, clinicians who identified a care recipient and wished to participate filled out a request form detailing their demands for ADL telemonitoring. An initial individual interview with the clinician was added before NEARS-SAPA reports were sent to them. This interview was specifically aimed at identifying the demands, facilitators of, and barriers to home care for this specific care recipient.

###### Use and Impact of ADL Telemonitoring

Qualitative data were similar to those collected in the alpha cycle (Textbox 2), with slight modifications in terms of time points and type of data collection: (1) follow-up focus group interviews with clinicians were replaced with individual interviews held every 4 months with clinicians following the installation of the sensors and (2) follow-up focus group interviews with administrators and managers were replaced with individual interviews at the end of the cycle.

In addition, after the first follow-up interview, 10 in-house questions specifically related to the ADL telemonitoring system and reported uses were sent to clinicians in the form of a web survey. Finally, 3 in-house questions were administered orally to care recipients regarding their perceptions of the usefulness of the telemonitoring system.

###### Facilitators of and Barriers to ADL Telemonitoring Deployment

Qualitative data were collected following procedures similar to those in the alpha cycle (Textbox 2), with the same slight modifications as those mentioned previously in terms of time points and type of data collection.

###### Usability and Acceptance of ADL Telemonitoring

Usability is defined as the effectiveness, efficiency, and satisfaction with which specified users achieve specified goals in particular technological environments [48]. Several measures were added to evaluate the usability of the system. During the initial interview, clinicians were asked whether they foresaw any facilitators of and obstacles to their intended use of the ADL telemonitoring system. During follow-up interviews, they were asked whether there were any barriers to and facilitators of their actual use of the telemonitoring system. A web survey comprising 7 questions from the System Usability Scale [49] was also sent to clinicians. The System Usability Scale is a reliable tool for measuring such usability, and the questions centered on the ADL telemonitoring system and reports. The survey also comprised an 11-question form built from the *perceived usefulness* and *ease of use* sections of the technology acceptance model–2 questionnaire [50]. The technology acceptance model–2 is an information systems theory that maps how users come to accept and use a technology.

To evaluate care recipients’ and caregivers’ level of acceptance of the presence of the sensors in their homes, they were asked to respond to an oral questionnaire composed of 8 questions developed by the research team.

###### Cost Estimation of ADL Telemonitoring

We collected metadata on each service provided to care recipients by the CIUSSS and CISSS and recorded them in Intégration CLSC. Intégration CLSC is a provincial ministry database containing personal information and providing data on service requests, users, and interventions concerning services delivered. The database is used to describe frontline services to ensure the quality and efficiency of health and social services. For this study, we examined the monthly count of services received by the care recipients within the time frame of 6 months preceding and 6 months following clinician access to ADL telemonitoring.

Throughout this project, we aimed to document the cost of implementing ADL telemonitoring in the public health care system by totaling direct, indirect, and intangible costs [51,52]. To do so, we documented the material costs (eg, number of devices used, lost material, server maintenance cost, software, licenses, material for fixtures, repairs and maintenance, internet services, and database and server maintenance). We also estimated the time invested by technicians and clinicians over the study period (eg, time for recruitment, needs analyses, installation, training, and technical support). This estimate was validated by the technicians and clinicians during group interviews in the postdeployment phase. The time invested was translated to cost using the common hourly rates for technicians and clinicians. Time dedicated exclusively to research purposes (eg, interviews about the usability of the system) was not included in the deployment costs.

#### Ethical Considerations

The beta cycle was approved by the Centre de recherche de l’Institut universitaire de gériatrie de Montréal research ethics board, mandated to be the evaluating committee in a multicenter procedure (CER VN 17-18-10). The same protocols and considerations as those in the alpha cycle were followed.

### Results

Results corresponds to the *Reflection and learning* stage of ADR. Data collection ended in late 2022. In total, the NEARS-SAPA was installed in 31 care recipients’ homes, and 34 clinicians were involved in the beta cycle. Data analyses for the beta cycle are nearing completion, and the results specific to each subobjective will be shared in the future. Three manuscripts currently being written and are expected to be published in 2024-2025.

## Discussion

### Expected Findings

The aging population and increasing demand for ADL support faced by social and health care services in many countries call for the deployment of innovative solutions to better meet the needs of older adults with cognitive deficits. In close collaboration with 3 public health care institutions in the province of Quebec, we conducted a study designed to arrive at an in-depth understanding of the potential of ADL telemonitoring to support older adults with cognitive decline in the context of a public health care system. More specifically, the objectives of the project were to (1) codevelop an ADL telemonitoring system that addresses the needs of all key stakeholders involved in home care services for older adults with cognitive deficits, including the older adults and their caregivers; (2) deploy ADL telemonitoring in the Quebec public health care system and identify the specific use cases; and (3) identify factors that could contribute to ensuring its long-term use in a real-world setting (ie, participants’ characteristics, demands, and use of telemonitoring; facilitators of and obstacles to its deployment; usability and acceptance; and estimated cost of the system). To achieve these objectives, we opted for an innovative research approach with ADR and engaged with all stakeholders involved in home care services throughout 2 iterative cycles of system codevelopment.

The publication of our protocol was part of the completion of the formalization of learning stage, the last ADR stage, comprising the identification of artifacts, generalization of learning, and dissemination. These 3 components will be addressed in this section.

### Identification of Artifacts

In this paper, we report the ADR protocol for the SAPA project and its related artifacts. As a form of ADR result, an artifact encompasses tangible or intangible elements, including objects, documents, processes, or outcomes, shaped by the ADR processes. These artifacts serve as tangible proof of the actions taken and the resulting benefits in the research context. The SAPA project has given rise to IT artifacts such as NEARS-SAPA (a web-based user interface supporting advanced data visualization and comparison tools) and NEARS-Hub (an Internet of Things infrastructure model) [47]. In addition, scientific and clinical artifacts such as the outcomes of the alpha cycle have been published in the form of case studies [15,45]. From data gathered within the beta cycle, our objective is to generate more artifacts, such as presenting comprehensive documentation and analyses of use cases along with factors supporting or limiting the deployment of the ADL telemonitoring system. This endeavor aims to contribute valuable scientific insights into the clinicians’ decision-making processes and deployment strategies that will further support the adoption and integration of ADL telemonitoring in real-life home care contexts.

### Generalization of Learning

This protocol paper also provides a thorough presentation of the entire project and its intricate phases. It illustrates how ADR principles could apply to the field of ADL telemonitoring, enabling stakeholders to be actively involved in developing technology that is useful, acceptable, and deployable in real-life environments. To our knowledge, a comprehensive ADR protocol spanning 2 cycles of development has not been published so far in the fields of smart environments and ADL telemonitoring. This is surprising as ADR offers a promising framework for developing practical technological solutions to complex problems, such as in health care services [35,37]. In this context, we present the detailed ADR protocol of this project as an outcome or artifact, enabling its replication in similar projects in the future and the generalization of our results.

Furthermore, the SAPA project and ADR protocol are innovative as they include and report the steps involved when ADL telemonitoring is deployed in real life. A recent umbrella review conducted by our team [31] revealed that few real-world deployment studies have been undertaken in the field of smart environments thus far. As an emerging area of research, published studies have primarily focused on the development and conceptualization of technological components, with only a limited number involving small-scale testing in a laboratory context. Consequently, there are limited published data on the deployment strategies required to integrate smart environments into the health care system or community [31], and our project was designed to fill this gap.

To our knowledge, we are the first to have codeveloped and deployed a system in iterative cycles of collaboration with all stakeholders involved in home care support in the context of a public social and health care system. Coconstruction and collaboration are at the heart of ADR principles. Other studies have reported the deployment of similar solutions, but the development or deployment were not carried out using a coconstruction approach in partnership with a health care system and did not consider how stakeholders wish to integrate telemonitoring information into their practice [14,49,53,54]. It has been shown that the codevelopment of technological solutions with all stakeholders and users is an important factor in ensuring that the deployment of sustainable technological solutions proceeds smoothly and leads to their long-term adoption [55]. Our protocol, centered on co-design, use cases, and deployment factors, is parallel to another recently published protocol reporting a clinical trial to document the effectiveness of an ADL telemonitoring platform via a quasi-experimental study [56]. In that study, more than 73 older adults will receive an active and assisted living–based multiservice assistance platform called HomeAssist. The platform includes ADL telemonitoring but also many applications designed for the older adults themselves to help them engage in their daily and social activities. Although the HomeAssist project has not yet been deployed in the home care services context at the time of publication, this type of project is key to advancing the field of ADL telemonitoring, for which very little rigorous experimental data exist thus far [31]. As for ADR procedures, this study illustrates how to realize various cycles of coconstruction and codeployment of a technology in close collaboration with stakeholders.

Although the SAPA project is innovative in terms of its vision and methodology, it operates on a relatively small scale. Therefore, it will be important to conduct larger-scale studies in the future. As a next step, we intend to conduct an evaluative study to determine the benefits of ADL telemonitoring. These benefits could include, for instance, effectiveness in terms of clinician productivity and work, older adults’ functional independence, use of other health services, and changes in the living environment, as well as benefits for the institutions in terms of cost-effectiveness [56]. To engage in these types of larger-scale evaluations, we recommend that research teams in the field of ADL telemonitoring rely on recognized models or frameworks of technology implementation and adoption that are well established in the digital health field. For example, the nonadoption, abandonment, scale-up, spread, and sustainability framework by Greenhalgh and Abimbola [55] enables anticipation of the challenges that may arise during the implementation of an eHealth system in a particular environment. As another example, the Clinical Adoption Framework [56] considers the micro-, meso-, and macrodimensions that influence the long-term adoption and sustainability of technological solutions. The use of such frameworks will ensure rigorous development of ADL telemonitoring supported by sophisticated data.

### Dissemination

Finally, in terms of dissemination, initial findings from the beta cycle were shared with the participating program managers, heads of services, and clinicians from the CISSS and CIUSSS. Ultimately, the comprehensive results and assessments will be shared with the scientific community, providers of home care services for older adults, managers, and policy makers. There has been discussion about progressing toward a third cycle of development. The primary objective of such a cycle would be to make NEARS-SAPA fully independent of the research team. Currently, the technology relies on support, supervision, and knowledge transfer from the research team for optimal functionality, posing a significant barrier to broader implementation—including integration into clinical processes—and cost-effectiveness.

### Conclusions

This paper reports the protocol for the SAPA project, which used an ADR framework to codevelop and deploy an ADL telemonitoring system in the Canadian public social and health care system. Through 2 ADR iterative cycles, the SAPA project made it possible to consider all stakeholders’ needs, expectations, and perceptions in the final telemonitoring strategies proposed as well as the imperatives related to the deployment of such solutions in real test environments. Future studies could follow this protocol and further develop some methodological aspects.

## Appendix

Multimedia Appendix 1 Description of specific methodological details for problem formulation, building, intervention, evaluation, and reflection and learning phase for both cycles.

## Abbreviations

ADL: activity of daily living
ADR: action design research
CISSS: centre intégré de santé et de services sociaux (integrated health and social services center)
CIUSSS: centre intégré universitaire de santé et de services sociaux (integrated university health and social services center)
CLSC: centre local de services communautaires (local community service center)
NEARS: Innovative Easy Assistance System
POC: proof of concept
SAPA: Soutien à l’autonomie des personnes âgées (Support for Seniors’ Autonomy program)
