# Supplementary material for: Codevelopment and Deployment of a System for the Telemonitoring of Activities of Daily Living Among Older Adults Receiving Home Care Services: Protocol for an Action Design Research Study
Source: JMIR Res Protoc. 2024 Feb 29;13:e52284. doi: 10.2196/52284 (PMC10940984; doi:10.2196/52284)
Supplement: Multimedia Appendix 1 [file resprot_v13i1e52284_app1.docx]

**Appendix 1 NEARS-SAPA Project**

**Supplementary Methodological Details**

| **Alpha cycle** | |
| --- | --- |
| 1. **Problem Formulation** | The perspectives of the following stakeholders were explored:   - administrators, who are responsible for managing budgets and resources within the SAPA program (i.e., the director, the associate director, the department head and the program managers of home care services) (n=2); - head of services (n=5); - clinicians (i.e., social and healthcare clinicians such as social workers, nurses, occupational and physical therapists) who conduct the evaluation, planning and delivery of services for home care (n=8); - care recipients (n=5); - caregivers (n=3).   The interviews were led by an experienced facilitator in qualitative research, and an observer took notes during focus groups to summarize the main ideas for approval by the group at the end of the sessions. All interviews and focus groups were audiotaped and transcribed. The verbatims were analyzed following a thematic content analysis method as described by Miles, Huberman and Saldana’s [60]. |
| 1. **Building** | **Sensors:** Four types of wireless sensors were used: passive infrared (PIR) sensors, magnetic contact sensors, smart electric switches, and water sensors.   - PIR sensors detect motion by receiving infrared radiation. They were used primarily to determine room occupation: after a PIR sensor was triggered in one room, the occupant was considered to occupy it for as long as the PIR sensor in another room was not triggered. PIR sensors were installed in the bedroom, kitchen, dining room, living room, entrance, and bathroom. One or two sensors were installed in most rooms depending on the size and layout of the room; - Magnetic contact sensors were used on the various cupboards or doors. A contact sensor was always installed on the front door and on the main drawer (or wardrobe door) used for clothing (e.g., underwear drawer). In the kitchen, the location and number of sensors varied depending on the layout and the occupant’s routine. The refrigerator, freezer, utensil drawer, kitchen cabinet (for pans, cups, plates), and one food storage cabinet, were equipped with sensors if possible and relevant, according to the occupant’s self-reported routine. - Smart electric switches were installed on the television and the microwave. Toaster, coffeemaker, and bedside lamp use could also be monitored if relevant, according to the occupant’s self-reported routine. - Water sensors were used in the bathroom sink, as well as the shower, to further help recognize activities related to hygiene. |
| 1. **Intervention** | **Recruitment procedures:**   - Inclusion criteria for care recipients were : 1) living with a loss of functional autonomy requiring home care services; 2) being 65 years of age and older or presenting a geriatric profile (i.e., multiple pathologies that require the intervention of a multidisciplinary team, functional decline with a risk of deterioration of health status or with a risk of hospitalization[61]). - If the case met the inclusion criteria, the clinician contacted the care recipient to briefly present the study and receive verbal consent to schedule a meeting at their home with a member of the research team. - When applicable, the care recipient’s caregiver was invited to this meeting and could participate in the research project as well. During that meeting, more information about the research project and the technology was given, and informed written consents were obtained. |
| 1. **Evaluation** | **Sub-units:**   - It should be noted that clinicians could change over the course of the project, due to leave, employee turnover, and organizational reallocation of resources. - Clinicians could recruit more than one care recipient and therefore participate in more than one subunit. - Many care recipients did not have a caregiver, or their caregivers were not available or interested in participating. Therefore, some subunits consisted only of a clinician and a care recipient.   **Data analysis:**   - Qualitative data from recordings of the focus groups and the individual interviews were transcribed by a person specialized in this type of work and checked by a member of the research team.   - Data analysis was performed using Miles, Huberman and Saldana’s method[60]. More precisely, three processes were used: memos, data codification, and matrix building. First, descriptive codes were created, which labelled units of text (words, sentences, paragraphs) that encompassed a distinct meaning. The coding grid emerged from the data. A brief definition of each code was developed as the coding process progressed. A list of codes, each supported by excerpts from interviews, was created. Codification was performed by research assistants and validated by two researchers (MC, NB). Second, matrices were used to compare data according to stakeholders. Matrix-building was done jointly by a researcher specialized in qualitative research (MC) and research assistants. - For quantitative data, descriptive analyses of average and standard deviation were used. |
|  | **Characteristics of care recipients:**With the care recipients’ consent, access to medical records was granted from January 2016 until the end of the participation. These data were extracted:  - Medical history; - Scores on the Mini-Mental State Evaluation (MMSE), Montréal Cognitive Assessment (MOCA). MMSE and MOCA are cognitive screening tests often used by health professionals in the detection of mild cognitive impairment and Alzheimer's disease [62, 63]. - Score on the Multi-client assessment tool (Outil d’Évaluation Multi-Clientèle: OEMC). The OEMC is an instrument used by all home care clinicians in the province of Québec, that facilitates and supports their own work, or multidisciplinary teams, in translating requests into needs, translating needs into services, and allocating available resources according to the needs of the care recipient [64]. It is composed of six forms, including a summary form that contains the intervention plan of the various actors involved with the care recipient. The OEMC also provides several demographic characteristics of the care recipient (i.e., age, gender, sex, education, medical diagnoses, living situation) as well as the Functional Autonomy Measurement System profile (*Système de mesure de l’autonomie fonctionnelle* : SMAF[65]). - The SMAF is a four-level rating scale used to quantify a subject's functional autonomy based on 29 functions among five types of activities, namely 1) ADL; 2) mobility; 3) communication; 4) mental functions; and 5) instrumental ADL. Following the completion of the SMAF by a clinician from home care services, individuals are classified according to the intensity and the type of services required to maintain their autonomy based on the information provided in the ISO-SMAF profiles. The ISO-SMAF classification consists of 14 homogeneous disability profiles characterized by a gradual progression in the severity of disabilities. - Metadata was also extracted from the medical records about each service provided by the health network for each care recipient respectively (from January 2016 until the end of their participation). This metadata detailed several variables for each service performed (i.e., type of act, reason for this act, its time and duration, the type of professional performing it, and its location).   Cognitive evaluation (Rey Auditory Verbal Learning[66], Mini-Geriatric Depression Scale[67], and D-KEFS Trail making test, Stroop test, and Tower Test [68]) and the Instrumental Activity Profile (IAP)[69] were completed by a research assistant with the care recipient. |
| 1. **Reflection and learning** | **Characteristics of subunits** The POC was deployed within one CLSC of CIUSSS 1 from 2017 to 2018.  Care recipient participants:   - Six potential home care recipients were identified, and the system was deployed in four care recipients’ homes.   - One installation was discontinued for a care recipient with paranoid personality disorder, as it was deemed potentially too stressful. - Care recipients were aged 73yo ±20 with an average of 11±7 years of schooling. All lived alone in a rented apartment. Two had family caregivers, two had neighbor caregivers and one had no caregiver. Their average MOCA score was 17±5. - Two participants had a diagnosis of multiple sclerosis. They were significantly younger (50yo±1) and scored predominant motor impairment on the SMAF (4 and 9). - Three other participants had various diagnoses of cognitive impairment (vascular cognitive impairment, subcortical dementia, and mild cognitive impairment). They were significantly older (90yo±2). - On the SMAF scale, one scored impairment limited to ADL (1) and the other two scored predominant motor impairment (5, 7). One participant had no diagnosed cognitive impairment despite having a low score on the MOCA scale (20). Her main medical condition was anxiety and risk of fall, and she scored impairment limited to ADL (1) on the SMAF scale.   Participating clinicians included three occupational therapists, three nurses, and two social workers. |
|  | **Documenting the implementation** **Obstacles:**   1. The technology used for the prototype was **suboptimal for some care recipients’ profiles**.    1. In one case, a care recipient with paranoid personality disorder and invasive thoughts about being robbed by a neighbor was referred to the project. At first, the clinician thought that the sensors could reassure him by showing that there was no intrusion in the home. However, following discussions with both the care recipient, their caregiver, and their clinician, it was decided that ADL telemonitoring should not be installed because the presence of the sensors could exacerbate his paranoid thoughts and feelings of being spied on.    2. Care recipients with important mobility challenges also appeared to benefit less from the ADL telemonitoring. Two participants with multiple lateral sclerosis were referred to the project because of their cognitive deficits. They had several support services (e.g., assistance with moving in and out of the bed, toilet, or bath). This limited the usefulness of the sensors as only a few actions related to ADL were performed without the presence of home care services. Both participants moved around their apartment in a wheelchair, therefore decreasing the monitoring efficiency and accuracy. Moreover, most objects they interacted with daily (e.g., clothes, food, dishware) were at their disposal on countertops instead of being stored away in drawers and cupboards, rendering contact sensors mostly useless. Because of mobility issues, the activities commonly associated with specific rooms were carried out in different locations. For instance, one participant slept, ate, and watched television on his adapted chair in the living room. Subsequently, it was decided to communicate to clinicians during the care recipient selection process that the technology was of limited usefulness for care recipients experiencing paranoid personality disorder or important mobility challenges. 2. Second, the alpha cycle protocol allowed us to better understand the population followed by the CSLC and to **refine the protocol for evaluating care recipients’ characteristics**.    1. The original protocol comprised many formal evaluations, including cognitive and ADL function testing to characterize the population. The clinicians mentioned that most care recipients who would benefit from ADL telemonitoring often do not respond well to formal evaluations. They added that several candidates would 1) be reluctant to be evaluated, 2) be unreliable in their answers or try to conceal their deficits, and 3) react with opposition and distrust if confronted with their personal challenges. This is especially true for those who fear being moved out of their home. Therefore, such a formal and confronting evaluation could lead to 1) refusal to participate or withdrawal from the study, 2) damage to the relationship between the care recipient and the clinician who suggested the project, and 3) increased intervention fatigue due to interaction with research staff (leading to more refusal of services offered by SAPA). As a result, clinicians might have more concerns and be reluctant to refer their care recipients to the telemonitoring program. For subsequent cycles, it was thus decided that formal evaluations with care recipients would be reduced as much as possible. 3. **The prototype was not ready for larger deployment**.    1. Installation required three hours of work at the care recipients’ home and several subsequent visits to adjust the sensors.    2. Data gathering and processing were too slow to support more than five apartments simultaneously.    3. Statistical output was limited, hard to interpret, and time-consuming to produce.    4. Several aspects of data processing lacked automation and had to be launched manually.    5. The prototype did not meet the web-security criteria for larger deployment in Québec health institutions. |
| **Beta cycle** | |
| 1. **Impacts of COVID-19** | This cycle took place between late-2018 and late-2022. Throughout 2019, a significant amount of time was devoted to technological developments. Recruitment of new participants started in late 2019. Therefore, the COVID-19 pandemic occurred as deployment for the beta cycle was just starting. The following limitations led to several modifications of the research protocol and monitoring system:   - Recruitment and deployment had to be delayed significantly in response to the emergency measures. - New challenges needed to be addressed to continue recruitment.   - First, several hygiene protocols had to be followed and contact with participants had to be reduced to the strict minimum.   - Second, clinicians were highly solicited to respond to health emergencies and had less availability for research – limiting our access to recruitment.   - Third, to prevent COVID-19 outbreaks, only authorized personnel could enter health care institutions. This meant that the research assistant could no longer install the telemonitoring platform on clinicians' computers and train them to use the system. |
| 1. **Building** | **System optimization:**   1. The **deployment of sensors was optimized**. Each system was prepared in advance in the research lab and sensors were identified, paired, and customized to match the home of the recipient. As a result, a shorter presence of the technician was required in the patient home for deployment (between 1½ and 2 hours, compared to 3 hours during the alpha cycle). 2. To facilitate **rapid data collection**, the Vera Z-wave home controller was replaced with a Raspberry Pi 3 linked to a USB Z-wave controller, offering several options for local data preprocessing as well as a number of other improvements. To modulate the services, the software components use a microservices architecture distributed around an event management platform in the form of streams. 3. Knowing that in an active and assisted living system, the collection of ambient information is central to activity recognition, software layers have been built to **automate the collection, maintenance, and processing of data**. This automation significantly speeds up the process and reduces the possibility of errors. 4. In case of Internet failure, data for sensors was **stored locally** until connectivity issues could be restored. 5. We improved the **robustness and reliability** of the information processing tools by distributing the processing over several calculation units and by automating local backups to compensate for service interruptions. 6. Achieving **compliance with the security and confidentiality** **standards of** the Québec Ministry of Health was a major achievement. We did so by providing a three-step technology solution. In other words, three complementary scales were developed for system security: 1) at the level of data collection, information was encrypted directly on the module installed in the care recipients’ home. On the different communication channels, attributes were encrypted in order to render the means of communication unreadable; 2) when transmitting data to our servers, we used the SSL communication protocol, for end-to-end encryption; and 3) a self-generated and self-signed certificate was set up to allow the clinicians to connect to the data visualization platform in a secure way, yet were restricted to data on their own care recipients. 7. Changes had to be made in response to **challenges raised by the pandemic**. Considering that health institutions could only be accessed by their own personnel, the research team could not gain access the clinicians’ office computers to install secure access to the NEARS-SAPA online and to demonstrate how to use the platform. Furthermore, as mentioned above, clinicians’ workload increased significantly. As such, clinicians and managers asked us to simplify the monitoring reports by describing how to read each infographic and highlighting key elements that could help in clinical decision-making. Given these obstacles, it was decided that the research team would consult the NEARS-SAPA platform and send clinicians monitoring reports in Portable Document Format (PDF) every two months, highlighting the trends and changes in ADL for each care recipient. This was deemed the most efficient way to share the monitoring data with clinicians in the context of the pandemic. |
| 1. **Intervention** | Based on prior experience, implementation strategies were put in place to improve the implementation process for the beta cycle:   1. With the involvement of managers from each health institution, it was decided that the project would **be presented during regular scheduled meetings of each targeted profession** (i.e., occupational therapists, physiotherapists, social workers, nurses, nutritionists, and respiratory therapists). During these presentations, clinicians were presented with: 1) personas and case applications for NEARS-SAPA; 2) a summary of the involvement required from them and their care recipient if they chose to participate, and; 3) the procedure for participating in the study. 2. Coordinators from different professional teams were asked to **promote the project to their colleagues**. 3. While it was not considered as an exclusion criterion, clinicians were warned about poor acceptance and possible adverse outcomes of ADL telemonitoring in individuals with **paranoid disorders**, especially if related to the fear of being observed and spied on. 4. Clinicians were also informed that ADL telemonitoring would be less accurate with individuals with **low mobility**, especially if they used a wheelchair and needed human assistance for transfers, dressing, washing themselves, and going to the bathroom. |
| 1. **Evaluation** | **Data analysis:** For qualitative data, the same analysis described in the alpha cycle was used. For quantitative data, descriptive analyses of average and standard deviation were used as well as regression, one-way analysis of variance and chi square depending on whether the variables were parametric or not. |
